# A scoring model for predicting prognosis of patients with severe fever with thrombocytopenia syndrome

**DOI:** 10.1371/journal.pntd.0005909

**Published:** 2017-09-21

**Authors:** Bei Jia, Xiaomin Yan, Yuxin Chen, Guiyang Wang, Yong Liu, Biyun Xu, Peixin Song, Yang Li, Yali Xiong, Weihua Wu, Yingying Hao, Juan Xia, Zhaoping Zhang, Rui Huang, Chao Wu

**Affiliations:** 1 Department of Infectious Diseases, Nanjing Drum Tower Hospital, Nanjing University Medical School, Nanjing, Jiangsu, China; 2 Department of Laboratory Medicine, Nanjing Drum Tower Hospital, Nanjing University Medical School, Nanjing, Jiangsu, China; 3 Department of Experimental Medicine and Medical Statistics, Nanjing Drum Tower Hospital, Nanjing University Medical School, Nanjing, Jiangsu, China; 4 Department of Intensive Care Units, Nanjing Drum Tower Hospital, Nanjing University Medical School, Nanjing, Jiangsu, China; Institute of Tropical Medicine, BELGIUM

## Abstract

Severe fever with thrombocytopenia syndrome (SFTS) is an emerging epidemic infectious disease caused by the *SFTS bunyavirus* (SFTSV) with an estimated high case-fatality rate of 12.7% to 32.6%. Currently, the disease has been reported in mainland China, Japan, Korea, and the United States. At present, there is no specific antiviral therapy for SFTSV infection. Considering the higher mortality rate and rapid clinical progress of SFTS, supporting the appropriate treatment in time to SFTS patients is critical. Therefore, it is very important for clinicians to predict these SFTS cases who are more likely to have a poor prognosis or even more likely to decease. In the present study, we established a simple and feasible model for assessing the severity and predicting the prognosis of SFTS patients with high sensitivity and specificity. This model may aid the physicians to immediately initiate prompt treatment to block the rapid development of the illness and reduce the fatality of SFTS patients.

## Author summary

Severe fever with thrombocytopenia syndrome (SFTS) is an emerging infectious disease caused by a novel *SFTS bunyavirus* (SFTSV) with an estimated high case-fatality rate. However, there is no specific antiviral therapy for SFTSV infection. Symptomatic treatment and supportive therapy are the most essential part of case management. It is very important for clinicians to identify critical patients at admission. In this study, we established a simple and feasible scoring system for assessing the severity and predicting the prognosis of SFTS patients with objective parameters. This model may help the physicians to perform intervention measures in advance, control the disease progression and improve the prognosis.

## Introduction

Severe fever with thrombocytopenia syndrome (SFTS) is an emerging infectious disease caused by a novel bunyavirus, which first emerged in China in 2005[1]. In 2009, the novel bunyavirus, designated as *SFTS virus* (SFTSV) or *Huaiyangshan virus*, was first isolated from patients with SFTS in China [2]. By the end of 2015, the disease was reported in 23 provinces in China, Japan and Korea [3–5]. Another similar *phlebovirus*, named *Heartland virus*, was reported as the cause of two cases of severe febrile illness with thrombocytopenia in Missouri, USA [6]. SFTS is characterized by fever, thrombocytopenia, leukocytopenia, elevated alanine and aspartate amino transferases, proteinuria and various other systemic manifestations including muscular symptoms, respiratory symptoms, gastrointestinal symptoms, neurological disorders and coagulopathy [7, 8]. It has become a significant public health threat with an estimated high case-fatality rate of 12.7% to 32.6% [5, 9, 10]. More importantly, previous research found that ribavirin may be effective more or less, but there is no specific antiviral therapy for SFTSV infection [11–15]. Symptomatic treatment and supportive therapy including intensive monitoring are the most essential part of case management [14]. Considering the higher mortality rate and rapid clinical progress of SFTS, supporting the appropriate treatment at early stage of disease and asking the doctor of intensive care unit (ICU) for consultation in time to SFTS patients is critical. Therefore, it is very important for clinicians to predict these SFTS cases who are more likely to have a poor prognosis or even more likely to decease.

Previous studies had reported many risk factors associated with fatal outcomes [5, 7, 8, 11, 16, 17]. For example, senior people is more likely to have a fatal clinical outcome [18]. Besides, the risk factors in terms of the clinical presentations includes acute lung injury or acute respiratory distress syndrome, central nervous system (CNS) symptoms, hemorrhagic manifestations, and disseminated intravascular coagulation [8, 17]. The risk factors regarding laboratory parameters include a higher serum viral load; the imbalance of cytokines and chemokines; decreased white blood cell counts (WBC) and platelets (PLT), higher aspartate aminotransferase (AST), alanine aminotransferase (ALT), lactate dehydrogenase (LDH), creatinine kinase (CK), alkaline phosphatase (ALP), gamma-glutamyl transpeptidase (GGT), blood urea nitrogen (BUN) level, and serum creatinine (sCr) and prolonged activated partial thromboplastin time (APTT)[5, 7, 8, 11, 16, 17]. However, their results were not consistent. A simple, practical and accurate prognostic scoring system will be more helpful to predict the prognosis of SFTS disease.

To our knowledge, only two models had been formed up today [8, 16]. However, their scoring criterion were not consistent. The model established by Xiong et al included a subjective parameter, which limited the clinical use of this model [8]. Further, two models do not have coagulation parameters such as APTT, which is a high risk factor for fatal outcomes reported in the previous studies [5, 8, 16].

So, the aim of our study was to establish a simple and feasible scoring system for assessing the severity and predicting the prognosis of SFTS patients with objective parameters, through fully understanding the clinical features and disease progress of SFTS.

## Methods

### Patients

A total of 142 SFTS patients who were admitted to Nanjing Drum Tower Hospital, Jiangsu, between October 2010 and July 2017 were enrolled in our study. In this study, all SFTS cases were diagnosed according to the following 2 conditions: (1) acute fever of > 38°C with thrombocytopenia (platelet (PLT) count <100×10^9^/L), (2) laboratory-confirmed SFTSV infection by using a certificated real-time polymerase chain reaction (RT-PCR) kit, performed by Jiangsu provincial center for disease control and prevention [15].

### Data collection

The demographic factors, date of illness onset, date of admission, date of death, disease outcome, clinical presentations, physical examination and laboratory parameters of these patients were retrospectively collected. The survival cases were followed for 30 days from onset of disease.

### Statistical analysis

Categorical variables were summarized as frequencies and proportions. Continuous variables with a normal distribution were described as means and standard deviations (SD), while the continuous variables with an abnormal distribution were shown as median and interquartile range (IQR). The chi-square test was used to compare categorical variables. Two-sample t-tests were used to compare the continuous variables with a normal distribution and Mann-Whitney U-tests were used to compare the continuous variables with an abnormal distribution between fatal and non-fatal cases. The risk factors for mortality in patients with SFTS were analyzed by binary logistic regression. Variables having *P* values <0.01 in the univariate analysis were used for a multivariate stepwise logistic regression analysis. Binary logistic regression analyses were used to develop the predictive models of death of SFTS. The predictive value of the model was evaluated by the receiver operating characteristic (ROC) curve. Differences between the AUCs were tested using the z-test. The probability cut-off points for the optimal combination of sensitivity and specificity were determined by the Youden index. The predictive model was validated by the standard diagnostic analysis of sensitivity, specificity and positive and negative likelihood ratio (LR). The patients were divided into four groups according to the interquartile range of the model (M) value of each patient. Kaplan-Meier survival analysis was used to compare the cumulative risk for death in the four groups, and the significance of difference was tested with the log-rank test. A *P* value < 0.05 was considered to be statistically significant. All statistical analyses were performed using SPSS (Statistical Package for the Social Sciences) version.22.0 software (SPSS Inc, Chicago, IL, USA) or SigmaPlot version 12.5 (Systat Software Inc., San Jose, CA, United States).

### Ethics statement

Patients all gave written consent to the participation in our study. The study was approved by the Ethics Committee of Nanjing Drum Tower Hospital.

## Results

### Patient description

A total of 142 SFTS patients were included in this study, including 33 fatal cases and 109 survival cases. The SFTS patients consisted of 67 males and 75 females. There was no difference in gender between fatal and survival cases. The median age of the fatal cases was significantly higher than that of the survival cases (65.2 vs 56.2 years, respectively; *P*<0.0001). The median time from the onset of illness to the hospital visit was 8 days (interquartile range [IQR], 6–9 days). There was no difference about the time from the onset of illness to the hospital between fatal and survival cases. 135 patients described a history of field exposure at mountainous or hilly areas, and 7 cases had contacted the blood or body fluid of index patient within 2–3 weeks of the disease onset.

### Clinical presentations and laboratory parameters in fatal and survival SFTS cases

The most frequently observed symptoms through the entire course and laboratory parameters on admission are shown in Tables 1 and 2. Among these commonly presented symptoms, high fever (T>39°C, 52% vs. 32.1%, *P* = 0.043), respiratory symptom (66.7% vs 35.8%, *P* = 0.002), neurologic symptoms (84.8% vs. 23.9%, *P*<0.0001), hemorrhagic manifestations (48.5% vs. 21.1%, *P* = 0.002) and hematuria (93.1% vs. 69.9%, *P* = 0.011) were significantly overrepresented in fatal cases. Compared to patients with SFTS who survived, the PLT, natural killer (NK) cell and serum albumin (Alb) were identified to be significantly lower in deceased patients on admission, whereas the viral load, red blood cell volume distribution width (RDW), B lymphocyte proportion, AST, ALT, ALP, GGT, direct bilirubin (Dbil), BUN, sCr, and LDH and CK values were significantly higher, and APTT and thrombin time (TT) were markedly extended in deceased cases.

**Table 1:** Clinical characteristics of hospitalized case-patients with confirmed severe fever with thrombocytopenia syndrome.

	NO.(%) or median (IQR)			
Characteristic	Total (n = 142)	Survival cases (n = 109)	Fatal cases (n = 33)	P value
Demographic feature				
Age (y)	58.3±12.0	56.2±12.0	65.2±9.3	<0.0001
Male sex	67 (47.2)	52 (47.7)	15 (45.5)	0.820
Duration day of disease course on admission	8 (6, 9)	8 (6, 9)	8 (7, 10)	0.241
General clinical manifestation				
Temperature>39°C	52 (36.6)	35 (32.1)	17 (52)	0.043
Fatigue	129 (90.8)	96 (88.1)	33 (100)	0.082
Headache	37 (26.1)	30 (27.5)	7 (21.2)	0.469
Myalgia	45 (31.7)	31 (28.4)	14 (42.4)	0.130
Lymphadenopathy	50 (35.2)	35 (32.1)	15 (45.5)	0.160
Gastrointestinal symptoms	129 (90.8)	98 (89.9)	31 (93.9)	0.720
Respiratory symptom	61(43.0)	39 (35.8)	22 (66.7)	0.002
Neurologic symptoms	54 (38.0)	26 (23.9)	28 (84.8)	<0.0001
Hemorrhagic manifestations	39 (27.5)	23 (21.1)	16 (48.5)	0.002
Proteinuria*	100 (82.0)	73 (78.5)	27 (93.1)	0.074
Hematuria*	92 (75.4)	65 (69.9)	27 (93.1)	0.011

*only available data were analyzed.

**Table 2:** Laboratory features of hospitalized case-patients with confirmed severe fever with thrombocytopenia syndrome by outcome on admission.

	Median (IQR)			
Laboratory tests	Total (n = 142)	Survival cases (n = 109)	Fatal cases (n = 33)	P value
Viral load (copies/mL)	5.35±1.75	4.92±1.60	6.89±1.30	<0.0001
WBC (10^9^ /L)	1.95 (1.35, 4.08)	2.00 (1.40, 4.25)	1.90 (0.65, 4.70)	0.619
Neutrophils count (10^9^ /L)	1.10 (0.70, 2.00)	1.10 (0.80, 2.00)	0.50 (0.35, 2.10)	0.794
Lymphocytes count (10^9^ /L)	0.70 (0.38, 1.30)	0.70 (0.40,1.15)	1.20 (0.30,1.70)	0.890
Monocyte count (10^9^ /L)	0.20 (0.10, 0.43)	0.20 (0.10, 0.45)	0.20 (0.10, 0.80)	0.586
Hb (g/L)	131.30±20.72	132.50±17.51	127.21±28.89	0.321
PLT (10^9^/L)	44.00 (29.50, 67.00)	54.00 (31.00, 69.00)	36.00 (23.00, 55.00)	0.010
RDW (%)	13.05 (12.60, 13.83)	13.00 (12.60, 13.60)	13.90 (12.50,14.30)	0.002
AST (U/L)	97.2 (52.28,169.10)	191.90 (112.60,409.15)	612.10 (321.50,1035.50)	<0.0001
ALT (U/L)	243.05 (113.10, 613.30)	92.20 (51.85, 149.05)	140.10 (70.10, 312.45)	0.005
ALP (U/L)	71.85 (52.55, 96.85)	65.70 (52.40, 85.05)	96.00 (59.15,128.15)	<0.0001
GGT (U/L)	46.70 (27.53, 134.23)	45.00 (27.35, 94.10)	145.40 (71.80, 179.55)	0.001
Dbil (mmol/L)	4.05 (2.68, 5.08)	4.00 (2.65,4.90)	4.20 (3.10, 12.00)	<0.0001
Alb (g/L)	33.10 (29.38, 36.08)	34.50 (30.20, 36.95)	28.70 (28.40,33.50)	0.008
BUN (μmol/L)	4.85 (3.28, 7.18)	4.60 (3.20,6.05)	8.30 (5.40, 15.30)	<0.0001
sCr (μmol/L)	64.00 (52.58, 77.15)	62.00 (51.15,71.50)	77.60 (70.50,194.00)	<0.0001
LDH (U/L)	833.00 (593.25, 1634.00)	685.00 (580.50, 1143.50)	1733.00 (928.00, 2887.50)	<0.0001
CK (U/L)	760.50 (310.50, 2135.25)	686.00 (302.00, 1732.50)	1600.00 (421.50, 5694.50)	0.005
CK-MB (U/L)	31.50 (17.00, 87.00)	30.00 (17.00, 65.00)	80.00 (12.50, 135.00)	0.251
APTT (s)	49.75 (38.30, 59.95)	44.50 (37.40, 54.85)	61.90 (58.30, 89.30)	<0.0001
TT (s)	26.75 (22.13, 49.03)	26.20 (20.45, 36.50)	100.00 (27.45, 111.10)	<0.0001
CD3^+^ cell (%)	59.05 (26.93, 71.13)	60.40 (44.00, 75.80)	20.40 (16.15, 47.95)	0.319
CD3^+^CD4^+^ cell percent (%)	24.20 (14.70, 39.85)	27.60 (20.00, 41.60)	8.60 (6.10, 26.20)	0.083
CD3^+^CD8^+^ cell percent (%)	31.10 (17.15, 37.80)	33.80 (25.10, 38.10)	7.80 (6.95, 21.85)	0.108
B cell percent (%)	16.60 (10.68, 21.85)	14.20 (10.05, 20.55)	58.20 (33.15, 68.40)	0.015
NK cell percent (%)	15.51±10.59	16.87±11.33	10.54±5.09	0.021

### Dynamic profile of laboratory findings in SFTS patients

**Fig 1:**
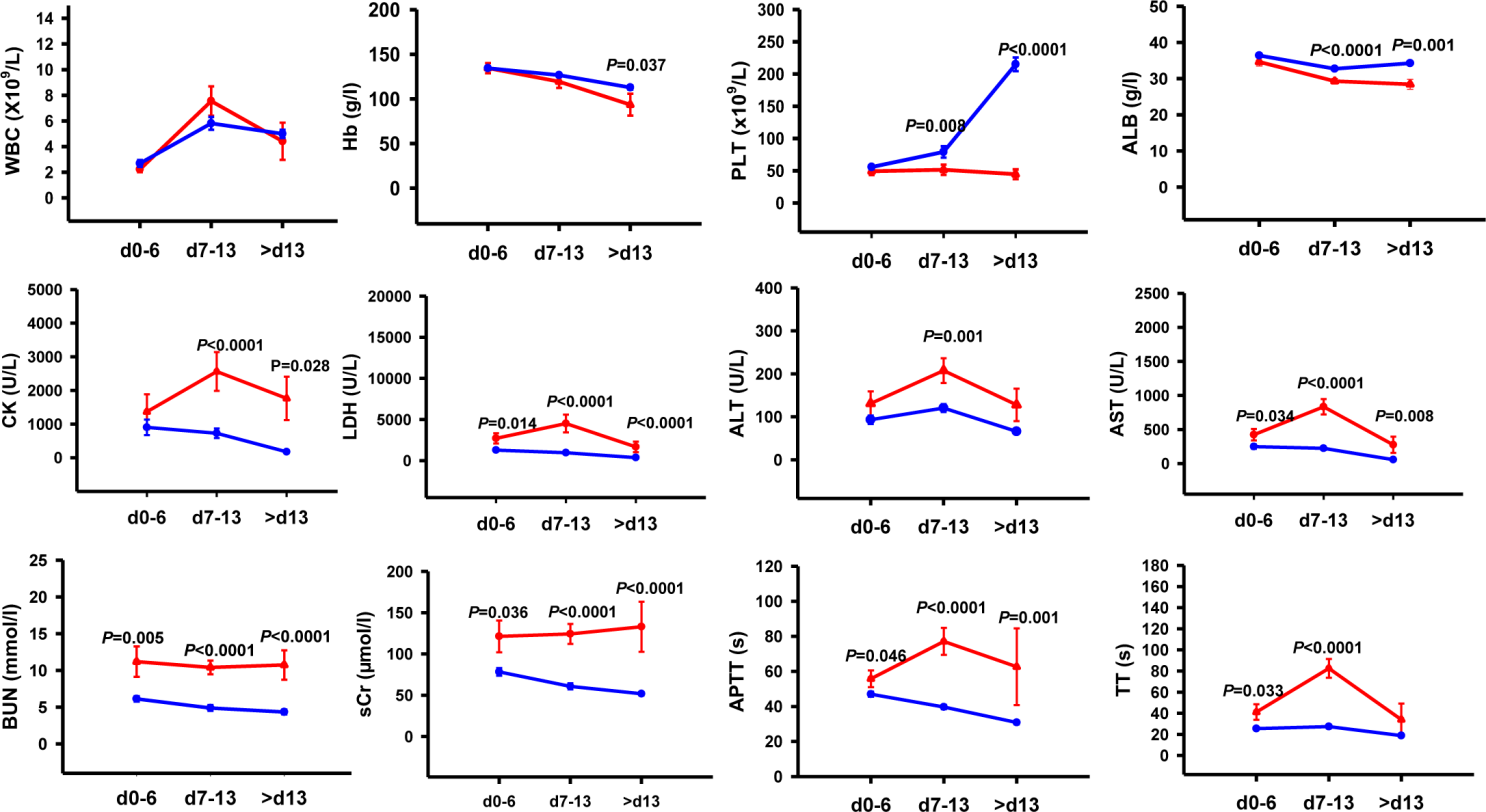
Changes in laboratory parameters at 3 periods in 142 patients with severe fever with thrombocytopenia syndrome. WBC, white blood cell; Hb, hemoglobin; PLT, platelet counts; Alb, serum albumin; LDH, lactate dehydrogenase; CK, creatinine kinase; AST, serum aspartate aminotransferase; ALT, serum alanine aminotransferase; TT, thrombin time; APTT, activated partial thromboplastin time; BUN, blood urea nitrogen; sCr, serum creatinine.

The dynamic changes of 12 clinical laboratory parameters, including hematological and biochemical parameters, were analyzed (Fig 1). The clinical course of SFTS infection has been divided into three distinct stages: fever stage (day 0–6), multiple organ dysfunction (MOD) stage (day 7–13) and convalescence stage (after day 13) [7, 19].

During the fever stage, thrombocytopenia, leukocytopenia, normal Alb and Hb, the elevated serum tissue enzymes level (ALT, AST, CK, and LDH), the prolonged APTT and TT were observed (Fig 1). The levels of AST and LDH were significantly higher in fatal cases in comparison with no-fatal cases (*P* = 0.034 and *P* = 0.014, respectively) (Fig 1). The APTT and TT was significantly longer in fatal patients when compared with survival cases (*P* = 0.046 and *P* = 0.033, respectively) (Fig 1). The BUN and sCr of the survival cases were in the normal range at the first stage, while they were outliers in the fatal cases. Compared to the no-fatal cases, the BUN and sCr were significantly higher in fatal cases (*P* = 0.005 and *P* = 0.036, respectively) (Fig 1).

During the MOD stage, as shown in Fig 1, an increased level of PLT in survivors but a decreased level of PLT in the patients who died was observed. The peripheral WBC counts were elevated, while Alb and Hb levels were diminished in both fatal cases and no-fatal cases during this stage. In SFTS patients who survived, the serum tissue enzymes (AST, CK, and LDH) began to decline slowly, but the ALT level rose slightly. However, all of them appeared to progressively rise during the MOD stage and were significantly higher in fatal cases. sCr level was increased in the fatal cases and was slowly decreased in no-fatal cases. The BUN level appeared to decline in two groups. The coagulation tests showed that the APTT and TT prolonged significantly in fatal cases, while both began to slowly shorten in survival cases (Fig 1). The Alb were markedly lower in fatal cases in comparison to survival cases (*P*<0.0001). Compared to survivors, the serum tissue enzymes (ALT, AST, CK, and LDH), BUN level and sCr level were significantly higher and the APTT and TT were pronouncedly longer in deceased cases (*P* = 0.001, *P*<0.0001, *P*<0.0001, *P*<0.0001, *P*<0.0001, *P*<0.0001, *P*<0.0001 and *P*<0.0001, respectively) (Fig 1).

After day 13, most clinical parameters converted to normal physical ranges in survivors. However, the fatal cases had the higher serum tissue enzymes and the renal function index (BUN and sCr), the lower level of PLT and Alb, and prolonged APTT at this stage, as shown in Fig 1.

### Risk factors for mortality

Univariate regression analyses of objective parameters for mortality of SFTS patients on admission were performed. The older age (OR, 1.084; 95% CI, 1.038–1.133; *P*< 0.0001), RDW (OR, 1.852; 95% CI, 1.144–2.998; *P* = 0.012), ALT (OR, 1.004; 95% CI, 1.001–1.008; *P* = 0.016), AST (OR, 1.003; 95% CI, 1.001–1.004; *P*< 0.0001), ALP (OR, 1.008; 95% CI, 1.003–1.013; *P* = 0.002), GGT (OR, 1.003; 95% CI, 1.000–1.006; *P* = 0.020), Dbil (OR, 1.032; 95% CI, 1.004–1.062; *P* = 0.025), Alb (OR, 0.871; 95% CI, 0.786–0.965; *P* = 0.008), BUN (OR,1.408; 95% CI, 1.236–1.602; *P*<0.0001), sCr (OR, 1.023; 95% CI, 1.013–1.033; *P*<0.0001), LDH (OR, 1.001; 95% CI, 1.001–1.002; *P*<0.0001), APTT (OR, 1.113; 95% CI, 1.069–1.159; *P*<0.0001), and TT (OR, 1.040; 95% CI, 1.024–1.055; *P*<0.0001) were the risk factors for fatal outcomes (Table 3). Variables having *P* values <0.01 in the univariate analysis were used for a multivariate stepwise logistic regression analysis. Multivariate regression analyses indicated that the older age (OR, 1.117; 95% CI, 1.046–1.194; *P* = 0.001), BUN level (OR, 1.277; 95% CI, 1.084–1.505; *P* = 0.003), and APTT (OR, 1.093; 95% CI, 1.041–1.148; *P*<0.0001) were the independent risk factors for fatal outcomes of SFTS patients (Table 3).

**Table 3:** Univariate and multivariate analyses of features associated with mortality in SFTS patients.

Factors	Univariate model		Multivariate model	
	OR (95% CI)	P value	OR (95% CI)	P value
Age (y)	1.084 (1.038–1.133)	<0.0001	1.117 (1.046–1.194)	0.001
PLT (10^9^ /L)	0.983 (0.966–1.001)	0.071		
RDW (%)	1.852 (1.144–2.998)	0.012		
ALT (U/L)	1.004 (1.001–1.008)	0.016		
AST (U/L)	1.003 (1.001–1.004)	<0.0001		
ALP (U/L)	1.008 (1.003–1.013)	0.002		
GGT (U/L)	1.003 (1.000–1.006)	0.020		
Dbil (μmol/L)	1.032 (1.004–1.062)	0.025		
Alb (g/L)	0.871 (0.786–0.965)	0.008		
BUN (mmol/L)	1.408 (1.236–1.602)	<0.0001	1.277 (1.084–1.505)	0.003
sCr (μmol/L)	1.023 (1.013–1.033)	<0.0001		
CK (U/L)	1.000 (1.000–1.001)	0.123		
LDH (U/L)	1.001 (1.001–1.002)	<0.0001		
APTT (s)	1.113 (1.069–1.159)	<0.0001	1.093 (1.041–1.148)	<0.0001
TT (s)	1.040 (1.024–1.055)	<0.0001		

### Clinical scoring model proposed for predicting SFTS mortality

As showed in the Table 3, multivariate regression analyses revealed that the older age, BUN level, APTT were the independent risk factors for fatal outcomes. Based on these independent predictors, a regression models were derived to predict fatal outcomes for SFTS patients. The model (M) is as follows:

$$M=\frac{1}{1+e^{-(-14.521+0.111\times Age+0.245\times BUN+0.089\times APTT)}}$$

**Fig 2:**
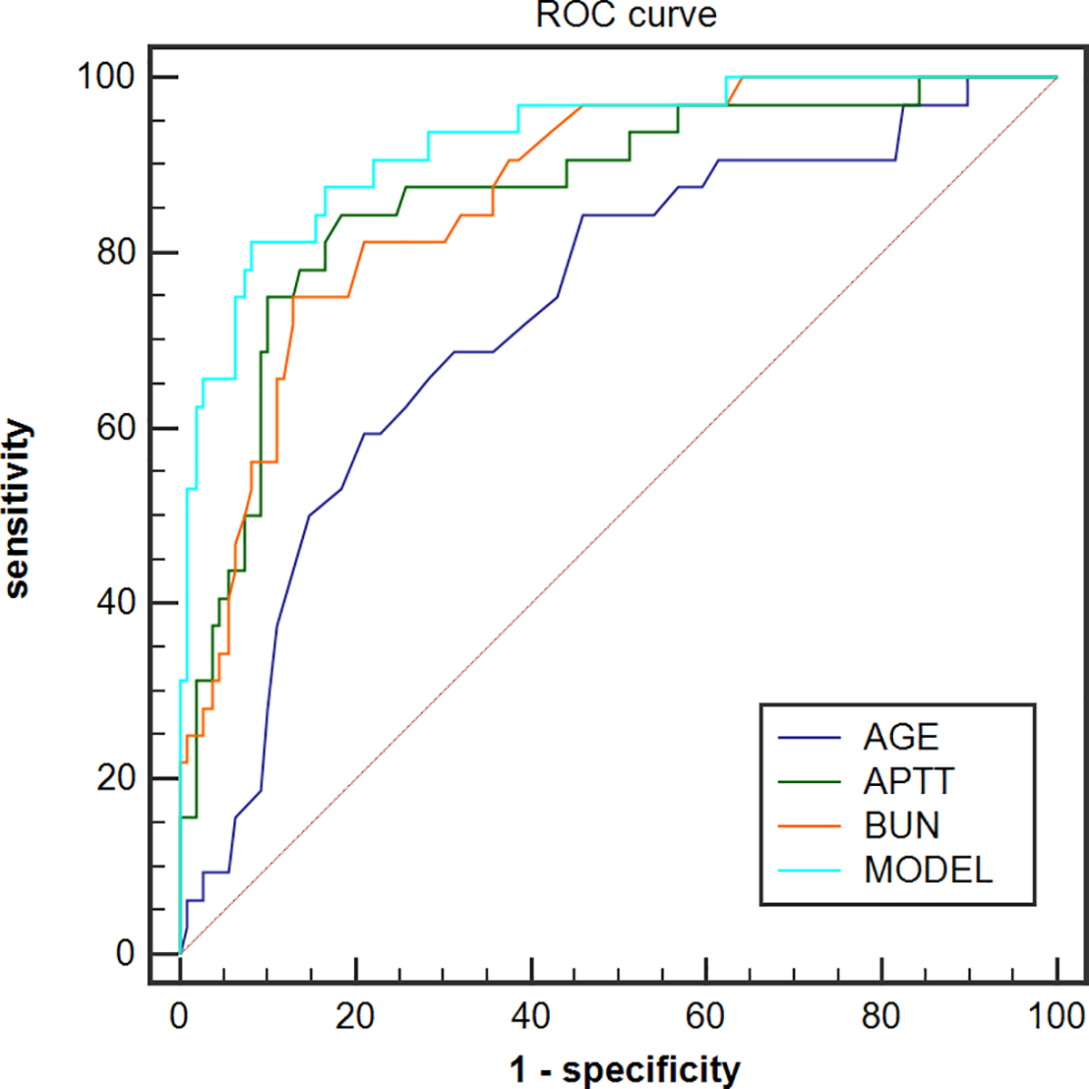
ROC curve for the prediction of mortality of SFTS patients.

ROC analysis was performed to compare the predictive value of the Model, Age, BUN and APTT. The cut-off values and area under ROC curve (AUCs) of these parameters for predicting death are included in Table 4. ROC curve is depicted in Fig 2. The model for predicting the mortality after infection with SFTSV showed an AUC of 0.927 (95% CI: 0.871–0.964, *P*<0.0001) with the optimal cut-off value of 0.28, which was higher than the AUCs of age (0.726, 0.645–0.797, *P* = 0.0001), APTT (0.870, 0.803–0.920, *P* = 0.025) and BUN (0.869, 0.801–0.920, *P* = 0.039). The model exhibited a significantly higher AUC in the prediction of death compared to the APTT (*P* = 0.025), age (*P* = 0.0001) and BUN (*P* = 0.039).

**Table 4:** Cut-off values, AUCs of different parameters for the prediction of mortality of SFTS patients with sensitivity and specificity.

Parameters	Cut-off value	AUCs (95%CI)	Sensitivity (%)	Specificity (%)	LR+	LR-
Age (y)	>66.00	0.726 (0.645–0.797)	57.58	78.90	2.73	0.54
APTT (s)	>51.90	0.870 (0.803–0.920)	84.85	81.65	4.62	0.19
BUN (mmol/L)	>7.32	0.869 (0.801–0.920)	75.00	87.16	5.84	0.29
Model	>0.28	0.927 (0.871–0.964)	81.25	91.74	9.84	0.20

**Fig 3:**
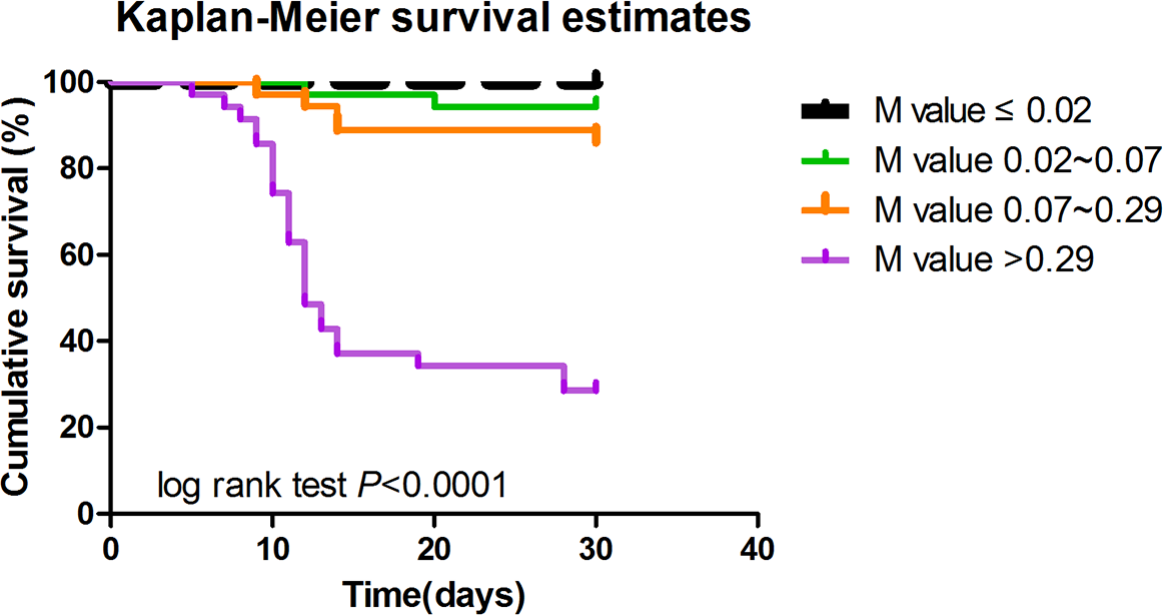
Survival curve of SFTS patients during 30 days’ follow-up based on risk score model.

**Fig 4:**
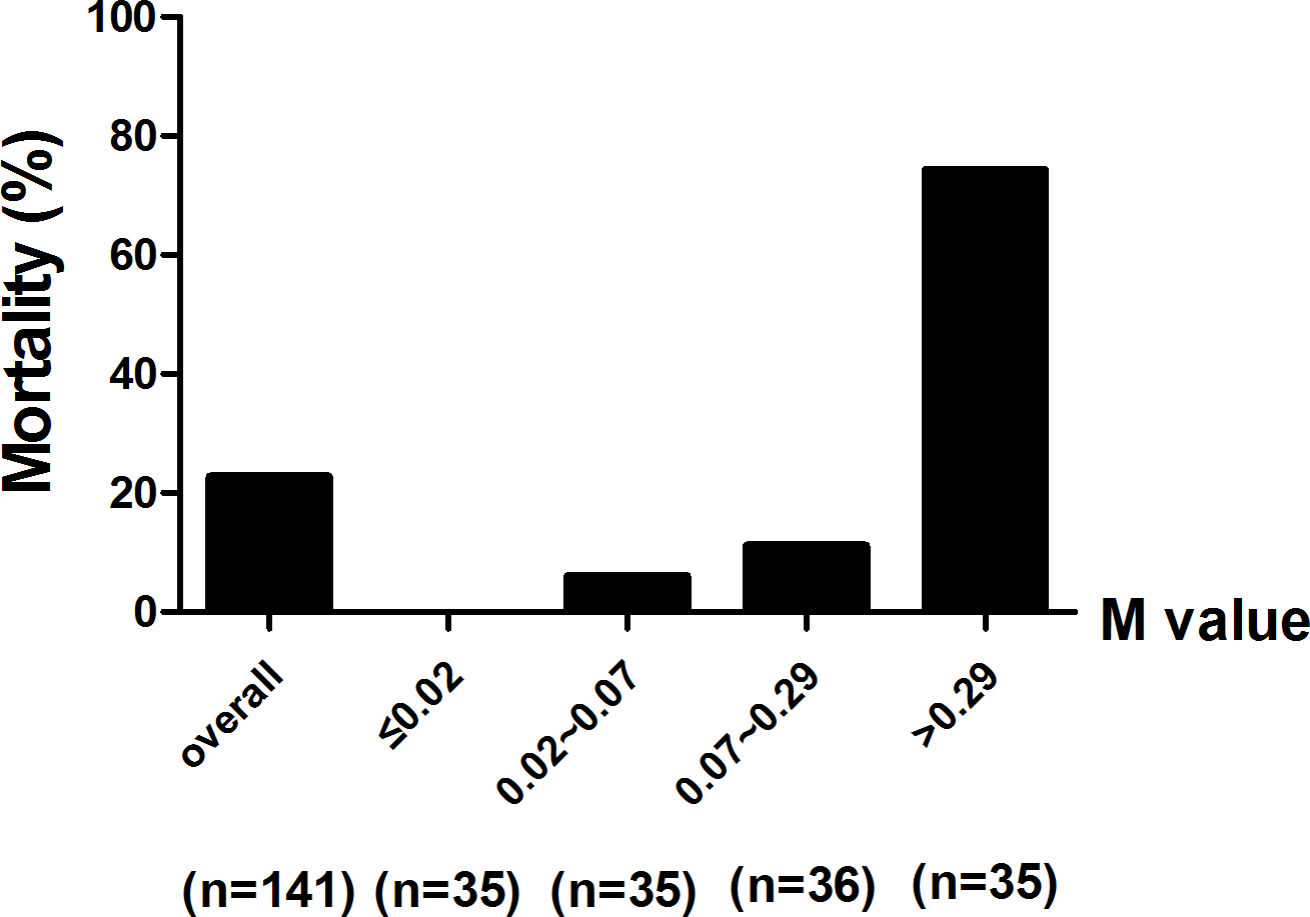
Mortality rate of the SFTS patients with different M value during 30 days’ follow-up. **M value = the model (M) value.** One patient was excluded due to incomplete data.

Kaplan-Meier survival analysis indicated that SFTS patients with the model scores >0.29 were much more likely to decease (log-rank test; χ2 = 82.20, *P*< 0.0001) during 30 days follow-up (Fig 3). M value of the model positively correlated with the fatality rate. The hospital mortality in different ranges of M value is shown in Fig 4. When the M value was greater than 0.29, the mortality was obviously higher than the overall mortality (74.3% vs 22.7%, respectively). No patient died with the M value less than 0.02. These findings suggested that the patients with M >0.29 were much more likely to decease after SFTSV infection.

## Discussion

The SFTS is an emerging infectious disease which has a mortality rate of up to 30% [2]. To better understand the disease progression of SFTS and identify the possible risk factors that are related to the fatal outcome of the patients, we systemically analyzed the risk factors between the fatal and survival SFTS cases in terms of demographic data, clinical symptoms and laboratory parameters in this retrospective study.

Fatal cases presented more severe clinical symptoms than non-fatal cases. High fever (T>39°C), respiratory symptoms and neurologic symptoms were significantly overrepresented in fatal cases. However, not all the fatal patients have these symptoms on admission. Some cases developed these symptoms afterwards.

The dynamic course of SFTS can be divided into three distinct stages of SFTS: fever, MOD, and convalescence stages according to the previous report [15]. In the present study, the serum tissue enzymes (ALT, AST, CK, and LDH), BUN level and sCr level were significantly higher and the APTT and TT were distinctly longer in fatal cases as compared with the non-fatal cases with SFTS in both fever and MOD stages. In the convalescence stages, the LDH, BUN and sCr level were also significantly higher and the APTT values were distinctly longer in fatal cases. Although the laboratory parameters were significantly different between fatal and non-fatal cases of SFTS during these stages, it is critical to identify the risk factors between the fatal and survival SFTS cases as soon as possible. The accurate prediction of the prognosis promptly may aid the clinicians to take the intervention measures in advance, control the disease progression and finally improve the prognosis for SFTS patients.

We compare the laboratory parameters between fatal cases and non-fatal cases of SFTS on admission. The biochemical, hematological parameters and coagulation variables on admission were shown that the PLT and Alb were significantly lower in deceased patients, whereas the viral load, B lymphocyte ratio, RDW, AST, ALT, ALP, GGT, Dbil, BUN, sCr, LDH and CK values were significantly higher, and the APTT and TT were markedly longer in deceased cases in comparison with the survivors. Thrombocytopenia, the decreased Alb an elevated AST, ALT, LDH, ALP, GGT, and sCr level were found to be the risk factors of patient mortality by univariate analysis. However, these parameters were not found to be statically significant after multivariate analysis in this study, which is not fully consistent with the previous studies [5, 8, 9, 12, 13, 16, 19].

We conducted univariate and multivariate analysis for objective indicators on admission which showed significant differences between the fatal and survival SFTS cases. Three identified independent risk factors are the older age, APTT and BUN, which were critical for predicting fatal outcome for SFTS patients. According to this study, the age in fatal cases was significantly higher in comparison to survival cases. Studies of demographic characteristics have also showed that age is a critical risk factor for death of SFTS patients [3, 5, 9, 18]. In our analysis, the APTT is a good factor to predict mortality for the SFTS cases, which was also found in the previous studies [5, 12, 20]. Overall, the APTT seemed to be the predominant factors closely related to hemorrhagic tendency. Based on the published reports and our own experience, most SFTS patients experienced MOD stage [5, 19]. Kidney function is currently recognized as an important indicator of MOD assessment, which is also important in the assessment of hemorrhagic fever with renal syndrome being caused by *Hantaan virus* which belongs to bunyavirus family [21]. One study also presented impaired renal and elevated BUN in an SFTSV-infected mouse model [22]. Renal function test, as a routine checkup, is simple and rapid, and is popular at all level of medical institutions now. BUN is a parameter that can indicate impairment of renal function. Our and others researches presented that BUN level as a key indicator for assessing kidney function was a high-risk factor for fatal outcome of SFTS patients [8, 12, 16, 17].

Finally, we established a risk model based on the three critical risk factors of age, APTT value and BUN level on admission, which showed relatively high predictive value with high, sensitivity and specificity (81.25% and 91.74%, respectively). The AUC of this model was 0.927, which was higher than the AUCs of the age, APTT and BUN. Furthermore, the Kaplan-Meyer analysis of overall survival was performed using the IQR of the M values on admission as the cut-off. The cumulative survival rate without an adverse outcome between the four groups was significantly different (log-rank test; *P*<0.0001). Survival rate was significantly lower in patients with the M value higher than 0.29 compared to patients with lower M values.

To our best knowledge, there were two models for predicting death for SFTS patients [8, 16]. Xiong et al proposed a scoring formula including viral load, neurologic symptoms, respiratory symptoms, and monocyte [8]. Indeed, a large amount of researches have shown that there was relatively higher serum viral load in patients who died than survivors [19, 23–25]. But viral load might not be a feasible predictor in clinical practice. In our cohort, the time of serum being sent to laboratory to detect SFTSV RNA varied among patients (5–28 days after onset of illness). Moreover, most hospitals, especially primary healthcare facilities, do not have the capacity to detect SFTSV RNA. Samples are transported to municipal centers for disease control and prevention (CDC) or provincial CDC of China for testing, which may lead to a delay in the key period for determining the condition of patients and giving treatment in time for clinicians. Moreover, neurological analysis was not a good parameter when scoring SFTS patients. Indeed, we showed central nervous system manifestation was an important risk factor which can predict death in SFTS patients independently, consistent previous reports [5, 8, 12, 19]. However, the description of clinical symptoms was affected by the subjectivity and experience of doctors. In addition, a study presented that neurological symptoms was not an independent risk factor for predicting death [16].

Another model established by Wang et al include baseline age, serum AST level and sCr level [16]. However, they did not include coagulation parameters in their model such as APTT which was a high-risk factor for fatal outcomes proposed in our and previous studies [5, 8, 12].

In addition, several findings proposed that the interval between onset and admission have an impact on the prognosis of the disease, while there was no difference between the deceased cases and survivors in our study [11, 26].

There are several limitations in this study. Firstly, the study is a retrospective study which would lead to bias the results. In addition, it is a single-center study and the sample size is relatively small. Thirdly, this research is lack of a validation group.

In conclusion, the present study provided important insights into the progression and prognosis of SFTS using age and objective laboratory indicators. The model including age, APTT value and BUN level, an inexpensive and easily calculated index, can predict fatal outcome of SFTS patients with relatively high accuracy. This model may aid the physicians to immediately initiate prompt treatment to block the rapid development of the illness and reduce the fatality of SFTS patients. However, the accuracy and sensitivity of this model needs to be further validated with large size of SFTS patients from multiple medical centers.

## Supporting information

S1 Table Clinical characteristics of patients with confirmed severe fever with thrombocytopenia syndrome. NA, not available.
(XLSX)

S2 Table Laboratory features of patients with confirmed severe fever with thrombocytopenia syndrome on admission. WBC, white blood cell; Hb, hemoglobin; PLT, platelet counts; RDW, red blood cell volume distribution width; ALP, alkaline phosphatase; GGT, gamma-glutamyl transpeptidase; Dbil, direct bilirubin; Alb, serum albumin; LDH, lactate dehydrogenase; CK, creatinine kinase; CK-MB, creatine kinase isoenzyme-MB; AST, serum aspartate aminotransferase; ALT, serum alanine aminotransferase; TT, thrombin time; APTT, activated partial thromboplastin time; BUN, blood urea nitrogen; sCr, serum creatinine. M value, the value of model; NA, not available.
(XLSX)

S3 Table Dynamic profile of laboratory findings in patients with confirmed severe fever with thrombocytopenia syndrome. WBC, white blood cell; Hb, hemoglobin; PLT, platelet counts; Alb, serum albumin; LDH, lactate dehydrogenase; CK, creatinine kinase; AST, serum aspartate aminotransferase; ALT, serum alanine aminotransferase; TT, thrombin time; APTT, activated partial thromboplastin time; BUN, blood urea nitrogen; sCr, serum creatinine; NA, not available.
(XLSX)
